# Thalamic nuclei in frontotemporal dementia: Mediodorsal nucleus involvement is universal but pulvinar atrophy is unique to *C9orf72*

**DOI:** 10.1002/hbm.24856

**Published:** 2019-11-07

**Authors:** Martina Bocchetta, Juan E. Iglesias, Mollie Neason, David M. Cash, Jason D. Warren, Jonathan D. Rohrer

**Affiliations:** ^1^ Dementia Research Centre, Department of Neurodegenerative Disease, UCL Queen Square Institute of Neurology University College London London UK; ^2^ Centre for Medical Image Computing, Department of Medical Physics and Biomedical Engineering University College London London UK; ^3^ Martinos Center for Biomedical Imaging Massachusetts General Hospital and Harvard Medical School Boston Massachusetts USA; ^4^ Computer Science and Artificial Intelligence Laboratory (CSAIL) Massachusetts Institute of Technology Boston Massachusetts USA

**Keywords:** frontotemporal dementia, magnetic resonance imaging, thalamic nuclei

## Abstract

Thalamic atrophy is a common feature across all forms of FTD but little is known about specific nuclei involvement. We aimed to investigate in vivo atrophy of the thalamic nuclei across the FTD spectrum. A cohort of 402 FTD patients (age: mean(*SD*) 64.3(8.2) years; disease duration: 4.8(2.8) years) was compared with 104 age‐matched controls (age: 62.5(10.4) years), using an automated segmentation of T1‐weighted MRIs to extract volumes of 14 thalamic nuclei. Stratification was performed by clinical diagnosis (180 behavioural variant FTD (bvFTD), 85 semantic variant primary progressive aphasia (svPPA), 114 nonfluent variant PPA (nfvPPA), 15 PPA not otherwise specified (PPA‐NOS), and 8 with associated motor neurone disease (FTD‐MND), genetic diagnosis (27 *MAPT*, 28 *C9orf72*, 18 *GRN*), and pathological confirmation (37 tauopathy, 38 TDP‐43opathy, 4 FUSopathy). The mediodorsal nucleus (MD) was the only nucleus affected in all FTD subgroups (16–33% smaller than controls). The laterodorsal nucleus was also particularly affected in genetic cases (28–38%), TDP‐43 type A (47%), tau‐CBD (44%), and FTD‐MND (53%). The pulvinar was affected only in the *C9orf72* group (16%). Both the lateral and medial geniculate nuclei were also affected in the genetic cases (10–20%), particularly the LGN in *C9orf72* expansion carriers. Use of individual thalamic nuclei volumes provided higher accuracy in discriminating between FTD groups than the whole thalamic volume. The MD is the only structure affected across all FTD groups. Differential involvement of the thalamic nuclei among FTD forms is seen, with a unique pattern of atrophy in the pulvinar in *C9orf72* expansion carriers.

## Graphical abstract

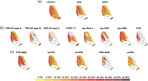

## 1 INTRODUCTION

The thalamus is the relay station of the brain, with so many different connections that it is virtually connected to all other brain regions. It is composed of several nuclei, each of them with specific connections and functional specialization (Table 1) (Behrens et al., 2003; Hale et al., 2015; Herrero et al., 2002; Kim et al., 2013; Lambert et al., 2017; Morel, 2007; Schmahmann, 2003; Zhang et al., 2008, 2010).

**Table 1:** Functions and connections of the thalamic nuclei. (Based on Schmahmann, 2003; Herrero, Barcia, & Navarro, 2002; Behrens et al., 2003; Lambert, Simon, Colman, & Barrick, 2017; Hale et al., 2015; Kim, Park, & Park, 2013; Zhang et al., 2008; Zhang, Snyder, Shimony, Fox, & Raichle, 2010; Iglesias et al., 2018)

Nuclei	Structural grouping	Functional grouping	Connections	Functions
AV	Anterior	Limbic	Entorhinal cortex, hippocampus (subiculum), amygdala, mammillary body, insula, frontal and temporal pole, anterior cingulate, retrosplenial cortex, orbitofrontal and medial prefrontal cortices	Learning and memory, spatial navigation, emotion, drive, motivation, anomia
LD	Lateral	Limbic	Mammillary body, cingulate, retrosplenial cortex, hippocampus (subiculum), entorhinal cortex, orbitofrontal and medial prefrontal cortices, amygdala	Limbic (learning and memory, emotional experience and expression, drive, motivation)
LP	Lateral	Associative	Somatosensory cortex, posterior parietal cortex, posterior cingulate, amygdala, hippocampus, medial and dorsolateral extrastriate cortex, medial parahippocampal cortex	Higher‐order somatosensory and visual–spatial integration (e.g., goal‐directed reaching, conceptual and analytical thinking)
VA	Ventral	Motor/limbic	Motor, premotor and supplementary motor cortex, prefrontal cortex, amygdala, accumbens, anterior cingulate, posterior parietal cortex	Complex behaviours, motor programming, limbic (learning and memory, emotional experience and expression, drive, motivation)
VLa	Ventral	Motor	Primary motor and premotor cortex, cerebellum (dentate), insula, frontal operculum, brainstem, ventral mesencephalon, pallidum	Motor
VLp	Ventral	Motor	Primary motor and premotor cortex	Motor, articulation and language, encoding and retrieval of verbal and nonverbal information
VPL	Ventral	Specific sensory	Somatosensory (superior frontal gyrus, insula, brainstem)	Somatosensory
VM	Ventral	Motor	Visual and motor cortex	Motor
Intralaminar	Intralaminar	Intralaminar	Motor cortex, striatum, basal ganglia (putamen), prefrontal cortex, brainstem, spinal cord, cerebellum [predominantly to subcortical instead of cortical structures]	Sensorimotor, limbic, cognitive; attention, arousal, consciousness, memory, autonomic drive, motivation, affective components of pain, sending attention‐specific sensory information to the striatum for conditional responses
Midline	Medial	Associative	Amygdala, orbital, medial, and dorsal prefrontal cortex, brainstem, spinal cord, cerebellum, periaqueductal grey	Anterograde and recognition memory, cognition, habituation, olfaction, vegetative and endocrine circadian activities, autonomic drive, sending attention‐specific sensory information to the striatum for conditional responses, processing motivational‐affective components of nociceptive information
MD	Medial	Associative/limbic	Temporal, dorsolateral and dorsomedial prefrontal, paralimbic regions (medial and orbital prefrontal cortex), amygdala, basal forebrain, olfactory and entorhinal cortex, motor and premotor cortex, anterior cingulate	Limbic, working memory, emotional regulation, behavior (inhibition, mood, perseveration), executive functions, vertical gaze (oculomotor control)
LGN	Posterior	Specific sensory	Occipital, parietal cortex ‐ primary visual cortex	Relay in the visual system
MGN	Posterior	Specific sensory	Temporal cortex ‐ primary auditory cortex	Relay in the auditory system
Pulvinar	Posterior	Associative/limbic	Prefrontal, posterior parietal, temporal, occipital and parietal cortex, hippocampus, amygdala, frontal operculum, anterior cingulate, insula, parahippocampal cortex, superior colliculi	Hallucination, visual attention, language, intramodality integration of somatosensory and visual information, pain appreciation, limbic, affective and psychotic symptoms

Thalamic atrophy is a common feature across all clinical, genetic, and pathological forms of frontotemporal dementia (FTD) (Bocchetta et al., 2018; Cardenas et al., 2007; Chow et al., 2008; Garibotto et al., 2011; Hornberger et al., 2012; Rohrer et al., 2015). FTD is a heterogeneous neurodegenerative disorder, with clinical symptoms spanning from behavioural changes to language and motor deficits. The main genetic causes are mutations in microtubule‐associated protein tau (*MAPT*), progranulin (*GRN*), and chromosome 9 open reading frame 72 (*C9orf72*) (Rohrer & Warren, 2011). The neuropathological abnormalities found in the brain in FTD fall into three main groups depending on the abnormal protein found in inclusions: tau, TDP‐43, and FUS (Lashley, Rohrer, Mead, & Revesz, 2015; Mackenzie & Neumann, 2016).

Given the extensive heterogeneity across the FTD spectrum, it is likely that the nuclei of the thalamus are differentially involved in the various forms of FTD and that some of the symptoms are related to the function of the nuclei affected. However, to date, no study has methodically looked at the specific thalamic nuclei in the FTD spectrum, with prior investigations focused on volumetry of the whole thalamus.

Due to recent advances in segmentation methods, it is now possible to measure individual thalamic nuclei *in vivo* on structural magnetic resonance (MR) scans (Iglesias et al., 2018). We therefore aimed to investigate the specific patterns of atrophy in the thalamic nuclei in a large cohort of FTD patients, to determine which nuclei are impaired across the different clinical, genetic, and pathological forms of FTD.

## 2 METHODS

We reviewed the UCL Dementia Research Centre FTD MRI database to identify 402 patients with a volumetric T1‐weighted MR scan passing standard quality control protocols and with a diagnosis of behavioural variant FTD (bvFTD) (Rascovsky et al., 2011), semantic variant primary progressive aphasia (svPPA), nonfluent primary progressive aphasia (nfvPPA) (Gorno‐Tempini et al., 2011), a primary progressive aphasia not otherwise specified (PPA‐NOS) (Harris et al., 2013), or FTD with associated motor neurone disease (FTD‐MND) (Table 2). One hundred‐four cognitively normal subjects, with a similar age to the patients and with a usable T1‐weighted MRI, were identified as controls. The study was approved by the local ethics committee and written informed consent was obtained from all participants.

**Table 2:** Demographic and clinical variables for the FTD patients and controls

Groups		*n*	Gender, male	Age at scan (years)	Disease duration (years)
Controls		104	44%	62.5 (10.4)	—
Clinical (*n* = 402)	FTD‐MND	8	63%	66.9 (4.3)	5.3 (2.9)
	bvFTD	180	68%	62.3 (8.0)	5.2 (3.2)
	nfvPPA	114	46%	67.5 (8.7)	4.4 (2.5)
	PPA‐NOS	15	67%	64.2 (6.1)	3.3 (1.7)
	svPPA	85	56%	63.9 (7.2)	4.8 (2.5)
Genetic^a^ (*n* = 73)	*C9orf72*	28	68%	62.8 (5.9)	5.5 (3.3)
	*GRN*	18	56%	62.0 (6.3)	3.1 (2.6)
	*MAPT*	27	63%	55.9 (7.5)	5.7 (3.2)
Pathological^a^ (*n* = 79)	TDP‐43 type A	15	60%	62.1 (5.8)	3.3 (1.7)
	TDP‐43 type B	3	67%	57.1 (7.7)	4.8 (2.7)
	TDP‐43 type C	20	65%	65.3 (7.3)	4.7 (2.8)
	FTDP‐17	7	71%	51.3 (5.8)	5.2 (3.1)
	tau‐Pick's	17	76%	59.7 (4.2)	4.4 (2.2)
	tau‐PSP	4	100%	76.9 (7.3)	5.1 (3.9)
	tau‐CBD	9	78%	61.8 (9.1)	4.5 (0.9)
	FUS	4	75%	45.7 (11.7)	3.0 (1.8)

*Note*: Values denote mean (*SD*) or *n* (%).

a Genetic and pathological cohorts are subsets of the overall clinical cohort with some overlap between the two (*n* = 20).

Seventy‐four patients had mutations in one of the FTD‐linked genes: 27 *MAPT*, 18 *GRN*, and 28 *C9orf72* carriers as well as one with a dual mutation in *GRN/C9orf72*, who was excluded from the individual group genetic analyses. For 81 patients, *post‐mortem* confirmation of the underlying neuropathology was available: FUS (*n* = 4), TDP‐43 type A (*n* = 15), TDP‐43 type B (*n* = 3), TDP‐43 type C (*n* = 20); tau with Pick's disease (*n* = 17), with PSP (*n* = 4), with CBD (*n* = 9), and due to FTDP‐17 (*n* = 7). We excluded from the pathological analysis 2 patients with tau‐GGT1 due to the small numbers. There was an overlap of 21 cases between the genetic groups and the pathological groups: 5 *GRN* had TDP‐43 type A, 6 *C9orf72* had TDP‐43 type A and 2 had TDP‐43 type B, 7 *MAPT* had FTDP‐17, and the patient with the dual *GRN/C9orf72* mutation had TDP‐43 type A.

Sociodemographic and clinical data are reported in Table 2. The mean age for the whole FTD group was 64.3 (*SD* 8.2) years with an average disease duration of 4.8 (2.8) years. There was no significant difference in age between FTD and controls (*p* = .067, *t*‐test), or for scanner type (*p* = .804, Chi square test), but there were more males in the FTD group than in the control group (59% vs. 44%, *p* = .006, Chi square test). Across the different clinical, genetic and pathological diagnoses, there was no difference for scanner type (*p* = .190, *p* = .615, and *p* = .053, Chi square test). There was a slightly significant difference in disease duration for the genetic (*p* = .018) and clinical groups (*p* = .042), but not for the pathological group (*p* = .084, ANOVA).

T1‐weighted MRIs were acquired from 1992 to 2018 with scanners from three different manufacturers: 231 on 1.5 T Signa MRI scanner (GE Medical systems, Milwaukee, WI, TR = 12 ms, TI = 650 ms, TE = 5 ms, acquisition matrix = 256 × 256, spatial resolution = 1.5 mm), 210 on 3 T Trio MRI scanner (Siemens, Erlangen, Germany, TR = 2,200 ms, TI = 900 ms, TE = 2.9 ms, acquisition matrix = 256 × 256, spatial resolution = 1.1 mm), and 65 on 3 T Prisma MRI scanner (Siemens, Erlangen, Germany, TR = 2000 ms, TI = 850 ms, TE = 2.93 ms, acquisition matrix = 256 × 256, spatial resolution = 1.1 mm).

Volumetric MRI scans were first bias field corrected and whole‐brain parcellated using the geodesic information flow (GIF) algorithm (Cardoso et al., 2015), which is based on atlas propagation and label fusion. Volumes of the whole thalamus and of its nuclei were subsequently segmented using a customised version of the module described in Iglesias et al., 2018, to accept the GIF parcellation as input for it. Based on anatomical subdivision, we combined the 52 original thalamic nuclei, and focused the analysis on the following 14 regions: anteroventral (AV), laterodorsal (LD), lateral posterior (LP), ventral anterior (VA), ventral lateral anterior (VLa), ventral lateral posterior (VLp), ventral posterolateral (VPL), ventromedial (VM), intralaminar, midline, mediodorsal (MD), lateral geniculate (LGN), medial geniculate (MGN) and pulvinar (Table 1 and Figure S1).

Left and right volumes were summed and expressed as a percentage of the total intracranial volumes (TIV), computed with SPM12 v6470 (Statistical Parametric Mapping, Wellcome Trust Centre for Neuroimaging, London, UK) running under Matlab R2014b (Math Works, Natick, MA) (Malone et al., 2015). All segmentations were visually checked for quality.

Statistical analyses were performed on thalamic volumes in SPSS software (SPSS Inc., Chicago, IL) v22.0, between control and FTD groups, using an ANOVA test adjusting for scanner type, TIV, gender, and age. Results were corrected for multiple comparisons (Bonferroni's correction) at *p* < .0035 (*p* = .05 divided by the 14 thalamic nuclei).

We performed a stepwise discriminant analysis between pairs of genetic, pathological, and clinical FTD subgroups for the thalamic nuclei and a second discriminant analysis for the whole thalamus.

## 3 RESULTS

**Figure 1:**
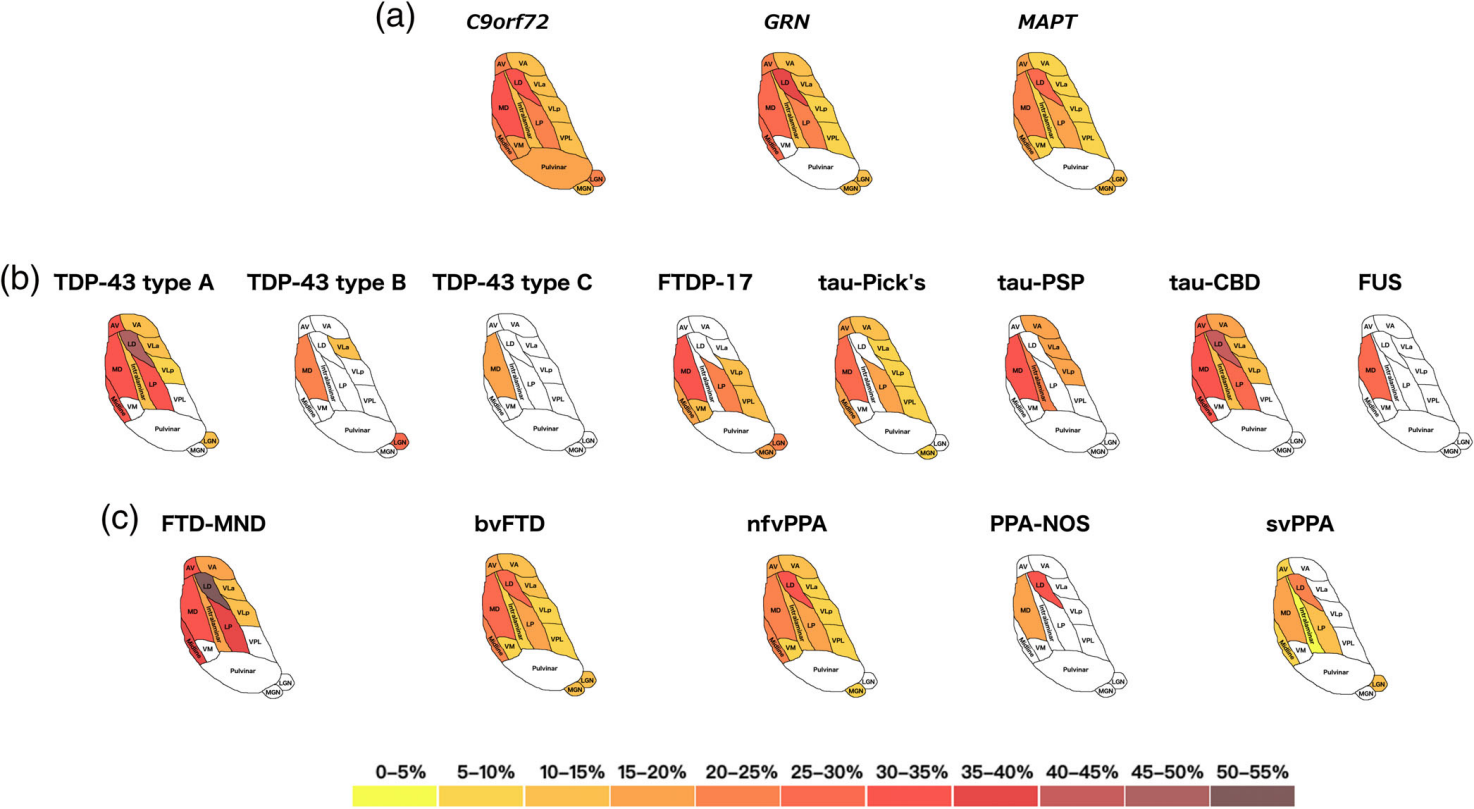
Pattern of atrophy in the thalamic nuclei in the (a) genetic; (b) pathological; and (c) clinical FTD groups. The cartoon is a schematic representation of an axial view of the thalamic nuclei and is not anatomically accurate. Colour bar denotes the % difference in volume from controls

Stratifying by genetics, the three groups showed significantly smaller thalamic nuclei than controls, except for VM for the *GRN* group and the pulvinar for both the *GRN* and *MAPT* groups. The pulvinar was only significantly smaller in *C9orf72* than controls (16% difference in volume, *p* < .0005 ANOVA) (Table 3 and Figure 1A). The MD and LD nuclei were particularly affected in all groups (21–32% and 28–38%, *p* < .0005), followed by AV (17–24%, *p* < .0005), midline nuclei (17–26%, *p* < .0005), and LP (15–23%, *p* < .0005). Both the LGN and MGN were also affected (*p* < .002), with the LGN smaller in the *C9orf72* group (20%) than the other groups (11%).

**Table 3:** Volumetric comparisons for the thalamic nuclei between the different genetic, pathological, and clinical subgroups and the controls

	*n*		AV	LD	LP	VA	VLa	VLp	VPL	VM	Intralaminar	Midline	MD	LGN	MGN	Pulvinar
**Genetic diagnosis**																
Controls	104	Mean	0.017	0.003	0.015	0.054	0.079	0.103	0.109	0.003	0.051	0.002	0.126	0.021	0.016	0.025
		*SD*	0.002	0.001	0.002	0.004	0.006	0.008	0.011	0.000	0.004	0.000	0.015	0.003	0.002	0.004
*C9orf72*	28	Mean	0.013	0.002	0.011	0.047	0.069	0.090	0.097	0.002	0.045	0.002	0.085	0.017	0.014	0.021
		*SD*	0.003	0.001	0.003	0.005	0.006	0.007	0.010	0.000	0.004	0.000	0.017	0.003	0.002	0.004
*GRN*	18	Mean	0.013	0.002	0.012	0.047	0.071	0.094	0.100	0.002	0.045	0.002	0.090	0.019	0.014	0.023
		*SD*	0.002	0.001	0.002	0.007	0.007	0.008	0.011	0.000	0.004	0.000	0.019	0.003	0.002	0.004
*MAPT*	27	Mean	0.014	0.002	0.013	0.050	0.073	0.096	0.102	0.002	0.046	0.002	0.099	0.019	0.014	0.025
		*SD*	0.003	0.001	0.002	0.005	0.006	0.009	0.012	0.000	0.005	0.000	0.019	0.004	0.002	0.003
Control	*C9orf72*	*P*‐value	**<.0005**	**<.0005**	**<.0005**	**<.0005**	**<.0005**	**<.0005**	**<.0005**	**<.0005**	**<.0005**	**<.0005**	**<.0005**	**<.0005**	**<.0005**	**<.0005**
		%	**24**	**34**	**23**	**13**	**12**	**12**	**11**	**15**	**13**	**22**	**32**	**20**	**12**	**16**
	*GRN*	*p*‐value	**<.0005**	**<.0005**	**<.0005**	**<.0005**	**<.0005**	**<.0005**	**.003**	.023	**<.0005**	**<.0005**	**<.0005**	**.002**	**<.0005**	.038
		%	**22**	**38**	**20**	**14**	**10**	**9**	**8**	8	**12**	**26**	**28**	**11**	**13**	8
	*MAPT*	*p*‐value	**<.0005**	**<.0005**	**<.0005**	**<.0005**	**<.0005**	**<.0005**	**<.0005**	**.001**	**<.0005**	**<.0005**	**<.0005**	**<.0005**	**<.0005**	.804
		%	**17**	**28**	**15**	**7**	**7**	**7**	**7**	**8**	**9**	**17**	**21**	**11**	**10**	0
**Pathological diagnosis**																
TDP‐43 type A	15	Mean	0.012	0.002	0.010	0.046	0.071	0.093	0.103	0.002	0.045	0.002	0.084	0.018	0.015	0.022
		*SD*	0.002	0.001	0.003	0.006	0.006	0.007	0.009	0.000	0.005	0.000	0.021	0.002	0.002	0.003
TDP‐43 type B	3	Mean	0.014	0.003	0.013	0.048	0.069	0.092	0.102	0.002	0.048	0.002	0.100	0.015	0.014	0.024
		*SD*	0.002	0.001	0.002	0.003	0.004	0.007	0.011	0.000	0.006	0.000	0.025	0.003	0.002	0.002
TDP‐43 type C	20	Mean	0.015	0.003	0.014	0.054	0.077	0.100	0.105	0.003	0.050	0.002	0.105	0.018	0.015	0.026
		*SD*	0.003	0.001	0.003	0.006	0.008	0.010	0.010	0.000	0.004	0.000	0.019	0.003	0.002	0.003
FTDP‐17	7	Mean	0.014	0.002	0.012	0.050	0.071	0.092	0.096	0.002	0.047	0.002	0.088	0.016	0.013	0.025
		*SD*	0.003	0.001	0.002	0.004	0.005	0.007	0.010	0.000	0.003	0.000	0.010	0.003	0.001	0.004
tau‐Pick's	17	Mean	0.014	0.002	0.013	0.049	0.072	0.096	0.104	0.003	0.049	0.002	0.089	0.021	0.014	0.027
		*SD*	0.004	0.001	0.003	0.007	0.008	0.010	0.008	0.000	0.005	0.000	0.014	0.002	0.002	0.003
tau‐PSP	4	Mean	0.013	0.002	0.011	0.044	0.064	0.083	0.091	0.002	0.040	0.002	0.087	0.016	0.013	0.026
		*SD*	0.001	0.001	0.001	0.003	0.007	0.009	0.006	0.000	0.002	0.000	0.015	0.002	0.002	0.006
tau‐CBD	9	Mean	0.012	0.002	0.011	0.044	0.067	0.090	0.105	0.003	0.046	0.002	0.088	0.020	0.015	0.026
		*SD*	0.003	0.001	0.002	0.004	0.006	0.007	0.006	0.000	0.004	0.000	0.012	0.004	0.002	0.004
FUS	4	Mean	0.014	0.003	0.013	0.050	0.074	0.098	0.106	0.003	0.049	0.002	0.093	0.021	0.014	0.028
		*SD*	0.002	0.001	0.003	0.008	0.009	0.011	0.013	0.000	0.005	0.001	0.025	0.004	0.003	0.004
Controls	TDP‐43 type A	*p*‐value	**<.0005**	**<.0005**	**<.0005**	**<.0005**	**<.0005**	**<.0005**	.012	.065	**<.0005**	**<.0005**	**<.0005**	**.003**	.013	*.004*
		%	**30**	**47**	**31**	**14**	**10**	**9**	6	8	**11**	**30**	**33**	**13**	8	*14*
	TDP‐43 type B	*p*‐value	.177	.589	.362	.017	**.003**	*.009*	.028	.057	.076	.234	**<.0005**	**.002**	.019	.999
		%	14	−3	10	11	**12**	*11*	6	8	6	4	**20**	**28**	11	4
	TDP‐43 type C	*p*‐value	.184	.286	.332	.738	.608	.319	.029	.142	.246	.071	**<.0005**	.023	.1	.106
		%	9	19	8	1	2	3	4	4	3	9	**16**	13	4	−3
	FTDP‐17	*p*‐value	.011	.043	**.001**	.023	.004	**.001**	**<.0005**	**.003**	*.006*	**<.0005**	**<.0005**	**<.0005**	**<.0005**	.57
		%	19	31	**22**	8	9	**11**	**12**	**12**	*9*	**17**	**30**	**22**	**15**	0
	tau‐Pick's	*p*‐value	**.002**	.01	**.001**	**<.0005**	**<.0005**	**.002**	**.003**	.081	.019	**<.0005**	**<.0005**	.482	**<.0005**	.024
		%	**15**	25	**15**	**10**	**8**	**7**	**5**	4	4	**17**	**29**	0	**9**	−6
	tau‐PSP	*p*‐value	.076	.436	.032	**.001**	**<.0005**	**<.0005**	*.005*	.015	**<.0005**	*.004*	**<.0005**	.095	.015	.401
		%	20	28	23	**19**	**19**	**19**	*17*	15	**22**	*30*	**31**	23	20	−2
	tau‐CBD	*p*‐value	**<.0005**	**<.0005**	**<.0005**	**<.0005**	**<.0005**	**<.0005**	.297	.603	**.002**	**<.0005**	**<.0005**	.784	.403	.358
		%	**28**	**44**	**29**	**18**	**14**	**12**	4	4	**11**	**30**	**30**	6	6	−2
	FUS	*p*‐value	.058	.956	.232	.048	.094	.162	.261	.606	.395	.016	**<.0005**	.936	.137	.045
		%	17	3	10	8	6	5	3	4	4	13	**26**	0	8	−12
**Clinical diagnosis**																
FTD‐MND	8	Mean	0.012	0.002	0.009	0.046	0.068	0.088	0.099	0.002	0.044	0.002	0.084	0.018	0.014	0.022
		*SD*	0.002	0.001	0.002	0.006	0.007	0.009	0.010	0.000	0.004	0.000	0.011	0.003	0.001	0.006
bvFTD	180	Mean	0.014	0.002	0.012	0.048	0.071	0.093	0.100	0.002	0.047	0.002	0.094	0.019	0.014	0.024
		*SD*	0.003	0.001	0.003	0.006	0.007	0.009	0.011	0.000	0.005	0.000	0.017	0.003	0.002	0.004
nfvPPA	114	Mean	0.014	0.002	0.012	0.049	0.072	0.094	0.103	0.002	0.046	0.002	0.097	0.019	0.015	0.025
		*SD*	0.003	0.001	0.003	0.006	0.007	0.009	0.010	0.000	0.005	0.000	0.016	0.003	0.002	0.004
PPA‐NOS	15	Mean	0.015	0.002	0.013	0.051	0.075	0.098	0.103	0.002	0.048	0.002	0.103	0.020	0.015	0.025
		*SD*	0.003	0.001	0.002	0.007	0.007	0.008	0.013	0.000	0.004	0.000	0.021	0.003	0.002	0.004
svPPA	85	Mean	0.015	0.003	0.013	0.053	0.076	0.099	0.105	0.003	0.049	0.002	0.105	0.019	0.015	0.027
		*SD*	0.003	0.001	0.003	0.005	0.007	0.009	0.011	0.000	0.005	0.000	0.018	0.003	0.002	0.003
Controls	FTD‐MND	*p*‐value	**<.0005**	**<.0005**	**<.0005**	**<.0005**	**<.0005**	**<.0005**	*.004*	.051	**<.0005**	**<.0005**	**<.0005**	.031	.01	.085
		%	**31**	**53**	**37**	**15**	**13**	**14**	*9*	8	**15**	**35**	**33**	15	11	11
	bvFTD	*p*‐value	**<.0005**	**<.0005**	**<.0005**	**<.0005**	**<.0005**	**<.0005**	**<.0005**	**<.0005**	**<.0005**	**<.0005**	**<.0005**	**<.0005**	**<.0005**	.099
		%	**17**	**28**	**18**	**12**	**10**	**9**	**9**	**8**	**9**	**22**	**25**	**11**	**11**	4
	nfvPPA	*p*‐value	**<.0005**	**<.0005**	**<.0005**	**<.0005**	**<.0005**	**<.0005**	**<.0005**	**.001**	**<.0005**	**<.0005**	**<.0005**	.014	**.001**	.747
		%	**16**	**31**	**18**	**10**	**9**	**8**	**6**	**8**	**10**	**22**	**23**	8	**7**	0
	PPA‐NOS	*p*‐value	*.008*	**<.0005**	.011	.137	.25	.219	.132	.266	.062	*.004*	**<.0005**	.333	.691	.492
		%	*13*	**31**	14	6	5	5	6	8	7	*17*	**18**	7	4	−1
	svPPA	*p*‐value	**<.0005**	**<.0005**	**<.0005**	.086	.054	*.009*	*.006*	.026	**.001**	**<.0005**	**<.0005**	**<.0005**	0.011	**.001**
		%	**9**	**22**	**11**	3	3	*4*	*4*	4	**4**	**9**	**17**	**10**	4	**−7**

*Notes*: Volumetric comparisons, expressed as % of total intracranial volume (TIV), are adjusted for age, gender, TIV, and scanner type. Bold represents a significant difference between groups after correcting for multiple comparisons. Percentage represents the volumetric difference between groups [(Column 1 − Column 2)/Column 1 × 100].

Stratifying by pathology, MD was the only nucleus affected in all subgroups (29–33% and 20% in TDP‐43 type B, *p* < .0005) and the only nucleus affected in FUS (26%, *p* < .0005) and TDP‐43 type C (16%, *p* < .0005) (Table 3 and Figure 1B). The LD was also particularly affected in TDP‐43 type A (47%, *p* < .0005) and tau‐CBD (44%, *p* < .0005). These two groups had the most nuclei affected followed by tau‐Pick's disease, with sparing of VPL, VM, and the pulvinar in each of these groups.

Stratifying by clinical diagnosis, the MD was affected in all subgroups, with FTD‐MND being the most affected (33%), and svPPA and PPA‐NOS the least (17–18%, *p* < .0005). The LD was also affected in all subgroups, especially in FTD‐MND (53%, *p* < .0005). The pulvinar was spared in all groups. FTD‐MND was the group with the smallest volumes overall, with PPA‐NOS the least affected group (Table 3 and Figure 1C).

Comparisons between the disease groups for each of the three analyses are reported in the Table S1.

We also repeated the above analyses in a purely sporadic cohort, excluding the 21 genetic cases. The results for the clinical and pathological subgroups showed a similar pattern of nuclei involvement despite a reduction in the sample sizes and therefore the statistical power. The sporadic FTD‐MND group still showed the highest volumetric differences from controls in the LP, MD, and midline nuclei (Table S2), and the sporadic TDP‐43 type A cases mainly showed volume differences in the MD, midline, LD, and LP (Table S3).

The volumetric comparisons between the FTD groups are reported in the Table S3.

Results of the discriminant analysis are shown in Table 4. Overall, the accuracy to correctly classify the groups was higher when using one or a combination of thalamic nuclei, than the whole thalamus. Among the genetic groups, the best classification was between *MAPT* and *C9orf72* using the pulvinar volumes, which correctly classified 70% of the *MAPT* carriers and 79% *C9orf72* (*p* < .0005). Among the pathological groups, 100% correct classification was obtained between FUS and tau‐PSP using VA, VLp, and intralaminar nuclei (*p* < .0005), between tau‐Pick's and tau‐PSP using LD, intralaminar, and LGN (*p* < .0005), between tau‐PSP and TDP‐43 type C using AV, VM, and intralaminar (*p* < .0005), and between TDP‐43 type A and TDP‐43 type B using LD, VLa, and midline nuclei (*p* = .001). Among clinical groups, classification accuracy was no better than using the whole thalamus (Table 4).

**Table 4:** Discriminant analysis between pairs of genetic, pathological and clinical FTD subgroups for the thalamic nuclei and the whole thalamus

		Thalamic nuclei				Whole thalamus	
		*p*‐Value	% CC	Predictors	Correlation	*p*‐Value	% CC
**Genetic diagnosis**							
*MAPT*	*GRN*	.046	89/50	VA	1	.955	
	*C9orf72*	<.0005	70/79	Pulvinar	1	.002	56/75
*GRN*	*C9orf72*	.042	33/82	LGN	1	.943	
**Pathological diagnosis**							
FUS	tau‐CBD	.021	50/89	LD	1	.900	
	FTDP‐17	.068	50/100	LGN	1	.776	
	tau‐Pick's	NA		NA		.992	
	tau‐PSP	<.0005	100/100	VA/VLp/Intralaminar	(−18.985/8.539/12.227)	.067	75/100
	TDP‐43 type A	.006	50/93	Pulvinar	1	.848	
	TDP‐43 type B	.076	100/0	LGN	1	.927	
	TDP‐43 type C	NA		NA		.997	
tau‐CBD	FTDP‐17	.006	100/71	Midline	1	.976	
	tau‐Pick's	.02	33/88	Midline/MD	(1.445/−1.022)	.945	
	tau‐PSP	.004	89/75	VPL	1	.074	89/25
	TDP‐43 type A	.006	67/87	VLa/Pulvinar	(−0.857/1.075)	.964	
	TDP‐43 type B	.003	100/33	Midline	1	.998	
	TDP‐43 type C	<.0005	56/90	Midline	1	.047	11/85
FTDP‐17	tau‐Pick's	.001	71/94	Intralaminar/LGN	(0.634/1.063)	.880	
	tau‐PSP	.003	86/100	Intralaminar/LGN	(1.378/0.975)	.038	100/75
	TDP‐43 type A	.017	57/87	VPL/Pulvinar	(−0.795/0.887)	.994	
	TDP‐43 type B	NA		NA		.956	
	TDP‐43 type C	.030	14/95	MD	1	.018	0/85
tau‐Pick's	tau‐PSP	<.0005	100/100	LD/Intralaminar/LGN	(−1.15/1.537/1.05)	.013	100/25
	TDP‐43 type A	<.0005	88/73	Pulvinar	1	.025	65/67
	TDP‐43 type B	.001	94/67	LGN	1	.979	
	TDP‐43 type C	<.0005	77/75	Intralaminar/MD/LGN	(0.694/−0.977/0.806)	.964	
tau‐PSP	TDP‐43 type A	.019	50/100	VPL	1	.937	
	TDP‐43 type B	.052	100/67	Intralaminar	1	.628	
	TDP‐43 type C	<.0005	100/100	AV/VM/Intralaminar	(−1.33/−1.093/2.51)	.004	25/95
TDP‐43 type A	TDP‐43 type B	.001	100/100	LD/VLa/Midline	(0.945/−1.418/1.097)	.971	
	TDP‐43 type C	<.0005	73/85	Midline/Pulvinar	(0.738/0.597)	.002	73/80
TDP‐43 type B	TDP‐43 type C	NA		NA		.939	
**Clinical diagnosis**							
bvFTD	nfvPPA	<.0005	84/36	AV/VM/Intralaminar/MGN	(0.807/1.091/−1.622/0.667)	.045	95/3
	PPA‐NOS	.031	100/0	MGN	1	.982	
	svPPA	<.0005	88/41	VA/Pulvinar	(0.782/0.433)	<.0005	92/25
	FTD‐MND	.002	100/0	LP	1	.981	
nfvPPA	PPA‐NOS	NA		NA		.991	
	svPPA	<.0005	76/47	VA	1	<.0005	84/41
	FTD‐MND	.004	100/0	LP	1	.007	100/0
PPA‐NOS	svPPA	NA		NA		.993	
	FTD‐MND	.002	87/63	Midline	1	.006	73/50
svPPA	FTD‐MND	<.0005	98/50	LP/Midline/Pulvinar	(0.482/0.452/0.543)	<.0005	98/13

*Notes*: “*p*‐Values” represents the Wills' Lambda significance for prediction accuracy, “% CC” represents the percentage of correctly classified subjects (where the first number refers to the reference group in the first column, and the second number to the group in the second column), while “Predictors” and “Correlation” represent respectively the nuclei included in the prediction models and their standardized canonical discriminant function coefficients.

## 4 CONCLUSIONS

In a large cohort of FTD patients we have shown that the MD is the only thalamic nucleus affected across all FTD groups. There is differential thalamic involvement among the FTD forms, with unique involvement of the pulvinar in *C9orf72* expansions carriers. Involvement of thalamic nuclei was more in the genetic forms of FTD than the sporadic forms with only MD involvement (and no other nuclei) in TDP‐43 type C and FUSopathies.

The MD is connected to several brain regions typically affected in FTD (Table 1), particularly prefrontal, temporal, and limbic areas that play a role in executive function as well as emotional and behavioural regulation. Prior small pathological studies have shown that the MD is specifically affected by chronic degenerative changes (e.g., neuronal depletion, gliosis, and astrocytosis) in FTD (in a single case with bvFTD (Radanovic et al., 2003) and in a group of ubiquitin‐positive cases (Mackenzie et al., 2006)).

The LD nucleus is also significantly smaller across the genetic and clinical groups: this is another nucleus with a key role in the limbic system, and is strongly connected to regions commonly affected across the FTD spectrum (Schmahmann, 2003) (Table 1).

Our findings of unique pulvinar atrophy in *C9orf72* expansion carriers are in line with the literature on imaging (Lee et al., 2014; Lee et al., 2016) and with pathological studies (Vatsavayai et al., 2016; Yang, Halliday, Hodges, & Tan, 2017) that have previously shown pulvinar involvement in *C9orf72* carriers: one histological study showed that almost all the neurons in the pulvinar had inclusions containing TDP‐43 and dipeptide repeat proteins (Vatsavayai et al., 2016). The pulvinar is a key region for limbic functions and intramodality integration of sensory information (Schmahmann, 2003) (Table 1). Atrophy in this nucleus can lead to altered processing of pain, hallucinations, and both affective and psychotic symptoms. This is in line with frequently reported clinical symptoms in *C9orf72* carriers that tend not to be found in other forms of FTD (Ducharme, Bajestan, Dickerson, & Voon, 2017; Fletcher et al., 2015). We also found that the LGN was particularly affected in *C9orf72*, an area previously linked to visual hallucinations which are a common feature of this genetic group (Ducharme et al., 2017; Kertesz et al., 2013).

We showed that by using the volume of a single thalamic nucleus or a combination of them, the accuracy in distinguishing between pairs of FTD subgroups was considerably higher than the accuracy in using the whole thalamus alone, especially for the genetic and pathological groups. This suggests that measuring individual thalamic nuclei rather than the whole thalamus may prove to be a better diagnostic biomarker for FTD, potentially as part of a wider set of volumetric measures.

Limitations of this study include the use of different scanners (three manufacturers, two different magnetic fields: 1.5 T and 3 T) with slightly different MRI sequence types. Even if we correct for scanner type and gender in the statistical model, we cannot completely remove some of the intrinsic heterogeneity due to these variables. However the algorithm operates at an internal resolution of 0.33 mm, which might compensate for the different native resolutions of the scans. Moreover, due to its unsupervised model for image intensities and to its Bayesian nature, this segmentation method is agnostic to the contrast of the MR images, and it is thus robust to the contrast changes between the scans acquired on different scanners. Whilst we used an automated method to extract the thalamic nuclei volumes, which is not as accurate as manual segmentation on dedicated MRIs or on brain tissue *post‐mortem*, we combined the 52 regions in the initial segmentation into 14 nuclei in order to decrease the effect of a less reliable delineation on T1‐weighted MRI. Furthermore, manual segmentation is extremely time‐consuming and labour‐intensive in such a large cohort. As there is no reliable measure of disease severity for FTD, and there is heterogeneity across its forms in the rate of disease progression, another limitation is the difficulty in characterising the level of disease severity between groups.

Further studies with longitudinal data and both diffusion‐weighted and functional MRI are needed to understand the differential involvement of thalamic nuclei over the course of the disease, and the changes in thalamic connectivity to other regions of the brain. Particularly important will be the investigation of presymptomatic mutation carriers in whom the earliest disease changes can be seen. However, this study has already highlighted both common and unique features of thalamic nuclei involvement across the FTD spectrum, adding to our understanding of the heterogeneity of this neurodegenerative illness.

## CONFLICT OF INTEREST

J.D.R. has been on a Medical Advisory Board for Wave Life Sciences and Ionis Pharmaceuticals.

## Supporting information


**Supplementary Table S1** Volumetric comparisons within the different genetic, pathological and clinical subgroups for the thalamic nuclei.
**Supplementary Table S2**. Volumetric comparisons for the thalamic nuclei between the clinical subgroups with sporadic FTD and the controls.
**Supplementary Table S3**. Volumetric comparisons for the thalamic nuclei between the pathological subgroups with sporadic FTD and the controls.
**Supplementary Figure S1**. Schematic representation of an axial view of the thalamic nuclei included in the analyses.

## Data Availability

The data that support the findings of this study are not publicly available due to ethical restrictions.

## References

[hbm24856-bib-0001] Behrens, T. E. , Johansen‐Berg, H. , Woolrich, M. W. , Smith, S. M. , Wheeler‐Kingshott, C. A. , Boulby, P. A. , … Matthews, P. M. (2003). Non‐invasive mapping of connections between human thalamus and cortex using diffusion imaging. Nature Neuroscience, 6(7), 750–757. 12808459 10.1038/nn1075

[hbm24856-bib-0002] Bocchetta, M. , Gordon, E. , Cardoso, M. J. , Modat, M. , Ourselin, S. , Warren, J. D. , & Rohrer, J. D. (2018). Thalamic atrophy in frontotemporal dementia—Not just a C9orf72 problem. Neuroimage Clinical, 18, 675–681. 29876259 10.1016/j.nicl.2018.02.019 PMC5988457

[hbm24856-bib-0004] Cardenas, V. A. , Boxer, A. L. , Chao, L. L. , Gorno‐Tempini, M. L. , Miller, B. L. , Weiner, M. W. , & Studholme, C. (2007). Deformation‐based morphometry reveals brain atrophy in frontotemporal dementia. Archives of Neurology, 64, 873–877. 17562936 10.1001/archneur.64.6.873 PMC2733361

[hbm24856-bib-0005] Cardoso, M. J. , Modat, M. , Wolz, R. , Melbourne, A. , Cash, D. , Rueckert, D. , & Ourselin, S. (2015). Geodesic information flows: Spatially‐variant graphs and their application to segmentation and fusion. IEEE Transactions on Medical Imaging, 34, 1976–1988. 10.1109/TMI.2015.2418298 25879909

[hbm24856-bib-0006] Chow, T. W. , Izenberg, A. , Binns, M. A. , Freedman, M. , Stuss, D. T. , Scott, C. J. , … Black, S. E. (2008). Magnetic resonance imaging in frontotemporal dementia shows subcortical atrophy. Dementia Geriatric Cognitive Disorders, 26, 79–88. 18617738 10.1159/000144028

[hbm24856-bib-0009] Ducharme, S. , Bajestan, S. , Dickerson, B. C. , & Voon, V. (2017). Psychiatric presentations of C9orf72 mutation: What are the diagnostic implications for clinicians? Journal of Neuropsychiatry and Clinical Neurosciences, 29(3), 195–205. 28238272 10.1176/appi.neuropsych.16090168

[hbm24856-bib-0010] Fletcher PD , Downey LE , Golden HL , Clark CN , Slattery CF , Paterson RW , Rohrer JD , Schott JM , Rossor MN , Warren JD . (2015) Pain and temperature processing in dementia: A clinical and neuroanatomical analysis. Brain 138(Pt 11):3360–3372. 26463677 10.1093/brain/awv276 PMC4620514

[hbm24856-bib-0011] Garibotto, V. , Borroni, B. , Agosti, C. , Premi, E. , Alberici, A. , Eickhoff, S. B. , … Padovani, A. (2011). Subcortical and deep cortical atrophy in frontotemporal lobar degeneration. Neurobiology of Aging, 32, 875–884. 19501427 10.1016/j.neurobiolaging.2009.05.004

[hbm24856-bib-0013] Gorno‐Tempini, M. L. , Hillis, A. E. , Weintraub, S. , Kertesz, A. , Mendez, M. , Cappa, S. F. , … Grossman, M. (2011). Classification of primary progressive aphasia and its variants. Neurology, 76(11), 1006–1014. 21325651 10.1212/WNL.0b013e31821103e6 PMC3059138

[hbm24856-bib-0014] Hale, J. R. , Mayhew, S. D. , Mullinger, K. J. , Wilson, R. S. , Arvanitis, T. N. , Francis, S. T. , & Bagshaw, A. P. (2015). Comparison of functional thalamic segmentation from seed‐based analysis and ICA. NeuroImage, 114, 448–465. 25896929 10.1016/j.neuroimage.2015.04.027

[hbm24856-bib-0015] Harris, J. M. , Gall, C. , Thompson, J. C. , Richardson, A. M. , Neary, D. , du Plessis, D. , … Jones, M. (2013). Classification and pathology of primary progressive aphasia. Neurology, 81(21), 1832–1839. 24142474 10.1212/01.wnl.0000436070.28137.7b

[hbm24856-bib-0016] Herrero, M. T. , Barcia, C. , & Navarro, J. M. (2002). Functional anatomy of thalamus and basal ganglia. Child's Nervous System, 18(8), 386–404. 10.1007/s00381-002-0604-1 12192499

[hbm24856-bib-0017] Hornberger M , Wong S , Tan R , Irish M , Piguet O , Kril J , Hodges JR , Halliday G . (2012) In vivo and post‐mortem memory circuit integrity in frontotemporal dementia and Alzheimer's disease. Brain 135(Pt 10):3015–3025. 23012333 10.1093/brain/aws239

[hbm24856-bib-0019] Iglesias, J. E. , Insausti, R. , Lerma‐Usabiaga, G. , Bocchetta, M. , Van Leemput, K. , Greve, D. N. , … Paz‐Alonso, P. M. (2018). A probabilistic atlas of the human thalamic nuclei combining ex vivo MRI and histology. NeuroImage, 183, 314–326. 30121337 10.1016/j.neuroimage.2018.08.012 PMC6215335

[hbm24856-bib-0020] Kertesz, A. , Ang, L. C. , Jesso, S. , MacKinley, J. , Baker, M. , Brown, P. , … Finger, E. C. (2013). Psychosis and hallucinations in frontotemporal dementia with the C9ORF72 mutation: A detailed clinical cohort. Cognitive and Behavioral Neurology, 26(3), 146–154. 24077574 10.1097/WNN.0000000000000008 PMC4090685

[hbm24856-bib-0021] Kim, D. J. , Park, B. , & Park, H. J. (2013). Functional connectivity‐based identification of subdivisions of the basal ganglia and thalamus using multilevel independent component analysis of resting state fMRI. Human Brain Mapping, 34(6), 1371–1385. 22331611 10.1002/hbm.21517 PMC6870335

[hbm24856-bib-0022] Lashley, T. , Rohrer, J. D. , Mead, S. , & Revesz, T. (2015). Review: An update on clinical, genetic and pathological aspects of frontotemporal lobar degenerations. Neuropathology and Applied Neurobiology, 41(7), 858–881. 26041104 10.1111/nan.12250

[hbm24856-bib-0023] Lambert, C. , Simon, H. , Colman, J. , & Barrick, T. R. (2017). Defining thalamic nuclei and topographic connectivity gradients in vivo. NeuroImage, 158, 466–479. 27639355 10.1016/j.neuroimage.2016.08.028

[hbm24856-bib-0024] Lee SE , Khazenzon AM , Trujillo AJ , Guo CC , Yokoyama JS , Sha SJ , Takada LT , Karydas AM , Block NR , Coppola G , Pribadi M , Geschwind DH , Rademakers R , Fong JC , Weiner MW , Boxer AL , Kramer JH , Rosen HJ , Miller BL , Seeley WW . (2014) Altered network connectivity in frontotemporal dementia with C9orf72 hexanucleotide repeat expansion. Brain 137(Pt 11):3047–3060. 25273996 10.1093/brain/awu248 PMC4208465

[hbm24856-bib-0025] Lee, S. E. , Sias, A. C. , Mandelli, M. L. , Brown, J. A. , Brown, A. B. , Khazenzon, A. M. , … Seeley, W. W. (2016). Network degeneration and dysfunction in presymptomatic C9ORF72 expansion carriers. NeuroImage: Clinical, 10(14), 286–297. 10.1016/j.nicl.2016.12.006 PMC5349617 28337409

[hbm24856-bib-0026] Mackenzie IR , Baker M , West G , Woulfe J , Qadi N , Gass J , Cannon A , Adamson J , Feldman H , Lindholm C , Melquist S , Pettman R , Sadovnick AD , Dwosh E , Whiteheart SW , Hutton M , Pickering‐Brown SM . (2006) A family with tau‐negative frontotemporal dementia and neuronal intranuclear inclusions linked to chromosome 17. Brain 129(Pt 4):853–867. 16401619 10.1093/brain/awh724

[hbm24856-bib-0027] Mackenzie, I. R. , & Neumann, M. (2016). Molecular neuropathology of frontotemporal dementia: Insights into disease mechanisms from postmortem studies. Journal of Neurochemistry, 138 Suppl 1, 54–70. 27306735 10.1111/jnc.13588

[hbm24856-bib-0028] Malone, I. B. , Leung, K. K. , Clegg, S. , Barnes, J. , Whitwell, J. L. , Ashburner, J. , … Ridgway, G. R. (2015). Accurate automatic estimation of total intracranial volume: A nuisance variable with less nuisance. NeuroImage, 104, 366–372. 25255942 10.1016/j.neuroimage.2014.09.034 PMC4265726

[hbm24856-bib-0029] Morel, A. (2007). Stereotactic atlas of the human thalamus and basal ganglia. New York: Informa Healthcare.

[hbm24856-bib-0030] Radanovic, M. , Rosemberg, S. , Adas, R. , Miranda, S. C. , Caramelli, P. , Caixeta, L. , & Nitrini, R. (2003). Frontotemporal dementia with severe thalamic involvement: A clinical and neuropathological study. Arquivos de Neuro‐Psiquiatria, 61(4), 930–935. 14762593 10.1590/s0004-282x2003000600008

[hbm24856-bib-0031] Rascovsky, K. , Hodges, J. R. , Knopman, D. , Mendez, M. F. , Kramer, J. H. , Neuhaus, J. , … Miller, B. L. (2011). Sensitivity of revised diagnostic criteria for the behavioural variant of frontotemporal dementia. Brain, 134, 2456–2477. 21810890 10.1093/brain/awr179 PMC3170532

[hbm24856-bib-0033] Rohrer, J. D. , & Warren, J. D. (2011). Phenotypic signatures of genetic frontotemporal dementia. Current Opinion in Neurology, 24, 542–549. 21986680 10.1097/WCO.0b013e32834cd442

[hbm24856-bib-0034] Rohrer, J. D. , Nicholas, J. M. , Cash, D. M. , van Swieten, J. , Dopper, E. , Jiskoot, L. , … Rossor, M. N. (2015). Presymptomatic cognitive and neuroanatomical changes in genetic frontotemporal dementia in the genetic frontotemporal dementia initiative (GENFI) study: A cross‐sectional analysis. Lancet Neurology, 14(3), 253–262. 25662776 10.1016/S1474-4422(14)70324-2 PMC6742501

[hbm24856-bib-0035] Schmahmann, J. D. (2003). Vascular syndromes of the thalamus. Stroke, 34(9), 2264–2278. 12933968 10.1161/01.STR.0000087786.38997.9E

[hbm24856-bib-0036] Vatsavayai SC , Yoon SJ , Gardner RC , Gendron TF , Vargas JN , Trujillo A , Pribadi M , Phillips JJ , Gaus SE , Hixson JD , Garcia PA , Rabinovici GD , Coppola G , Geschwind DH , Petrucelli L , Miller BL , Seeley WW . (2016) Timing and significance of pathological features in C9orf72 expansion‐associated frontotemporal dementia. Brain 139(Pt 12):3202–3216. 27797809 10.1093/brain/aww250 PMC5790143

[hbm24856-bib-0037] Yang, Y. , Halliday, G. M. , Hodges, J. R. , & Tan, R. H. (2017). von Economo neuron density and thalamus volumes in behavioral deficits in Frontotemporal dementia cases with and without a C9ORF72 repeat expansion. Journal of Alzheimer's Disease, 58(3), 701–709. 10.3233/JAD-170002 28482638

[hbm24856-bib-0038] Zhang, D. , Snyder, A. Z. , Fox, M. D. , Sansbury, M. W. , Shimony, J. S. , & Raichle, M. E. (2008). Intrinsic functional relations between human cerebral cortex and thalamus. Journal of Neurophysiology, 100(4), 1740–1748. 18701759 10.1152/jn.90463.2008 PMC2576214

[hbm24856-bib-0039] Zhang, D. , Snyder, A. Z. , Shimony, J. S. , Fox, M. D. , & Raichle, M. E. (2010). Noninvasive functional and structural connectivity mapping of the human thalamocortical system. Cerebral Cortex, 20(5), 1187–1194. 19729393 10.1093/cercor/bhp182 PMC2852505

