# Supplementary material for: Thalamic nuclei in frontotemporal dementia: Mediodorsal nucleus involvement is universal but pulvinar atrophy is unique to C9orf72
Source: Hum Brain Mapp. 2019 Nov 7;41(4):1006–16. doi: 10.1002/hbm.24856 (PMC7267940; doi:10.1002/hbm.24856)
Supplement: Supplementary file 1 — Supplementary Table S1 Volumetric comparisons within the different genetic, pathological and clinical subgroups for the thalamic nuclei. Supplementary Table S2. Volumetric comparisons for the thalamic nuclei between the clinical subgroups with sporadic FTD and the controls. Supplementary Table S3. Volumetric comparisons for the thalamic nuclei between the pathological subgroups with sporadic FTD and the controls. Supplementary Figure S1. Schematic representation of an axial view of the thalamic nuclei included in the analyses. [file HBM-41-1006-s001.docx]

**Supplementary Figure. Schematic representation of an axial view of the thalamic nuclei included in the analyses.**

**
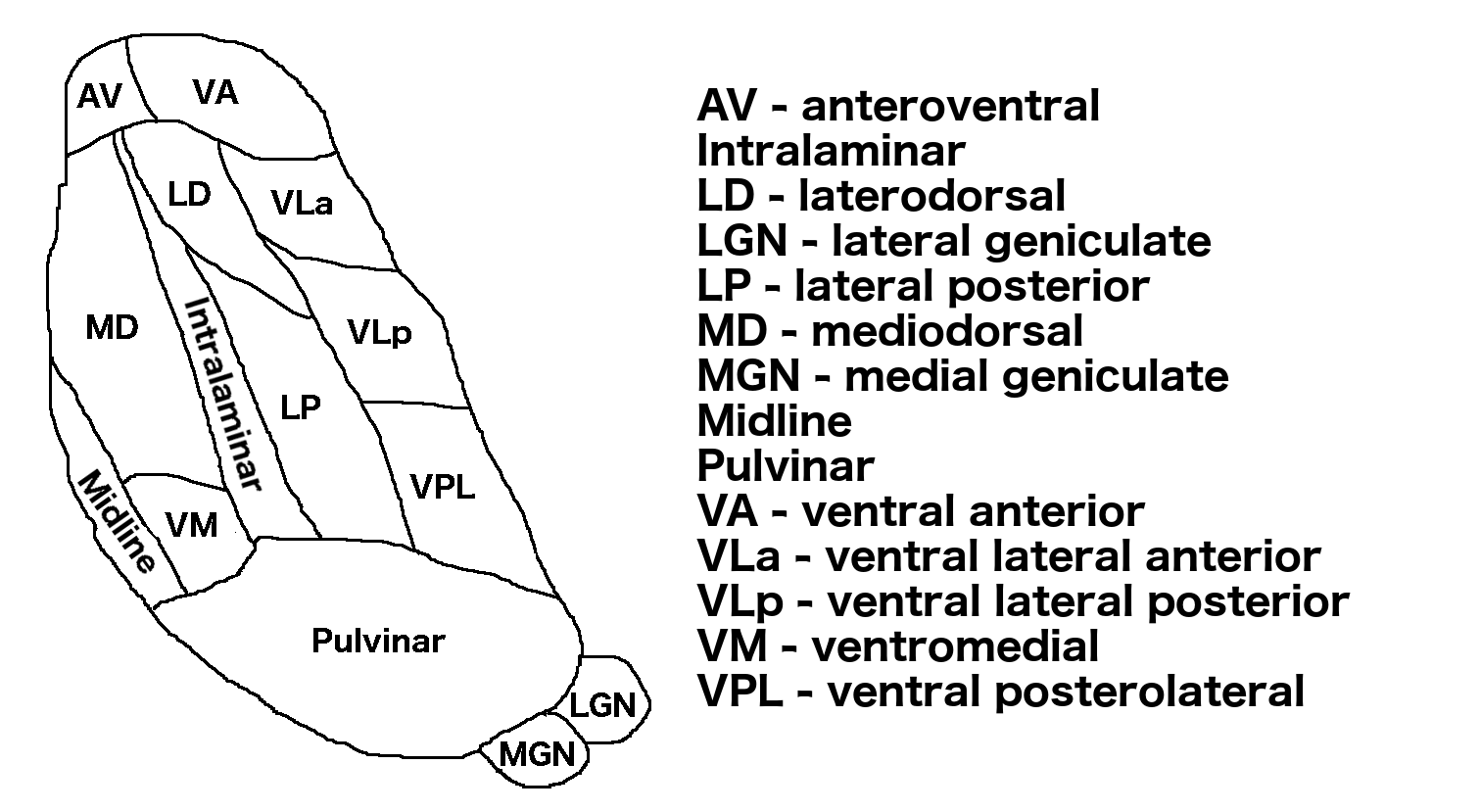
**

**Supplementary Table 1. Volumetric comparisons within the different genetic, pathological and clinical subgroups for the thalamic nuclei.** Volumetric comparisons are adjusted for age, gender, and scanner type. Bold represents a significant difference between groups after correcting for multiple comparisons. % represents the volumetric difference between groups [(Column 1 – Column 2)/Column 1 * 100].

| **Genetic diagnosis** | | | | | | | | | | | | | | | | |
| --- | --- | --- | --- | --- | --- | --- | --- | --- | --- | --- | --- | --- | --- | --- | --- | --- |
|  |  |  | **AV** | **LD** | **LP** | **VA** | **VLa** | **VLp** | **VPL** | **VM** | **Intralaminar** | **Midline** | **MD** | **LGN** | **MGN** | **Pulvinar** |
| ***MAPT*** | ***GRN*** | *p-value* | 0.243 | 0.201 | 0.159 | 0.098 | 0.564 | 0.921 | 0.815 | 0.571 | 0.891 | 0.684 | 0.333 | 0.673 | 0.609 | 0.124 |
|  |  | % | 6 | 13 | 6 | 7 | 3 | 2 | 2 | 0 | 2 | 11 | 9 | 1 | 4 | 7 |
|  | ***C9orf72*** | *p-value* | 0.133 | 0.264 | 0.039 | 0.168 | 0.058 | 0.102 | 0.306 | 0.262 | 0.509 | 0.915 | 0.018 | 0.069 | 0.588 | **<0.0005** |
|  |  | % | 8 | 9 | 10 | 6 | 6 | 6 | 5 | 8 | 3 | 5 | 14 | 10 | 3 | **15** |
| ***GRN*** | ***C9orf72*** | *p-value* | 0.857 | 0.773 | 0.655 | 0.660 | 0.253 | 0.164 | 0.242 | 0.110 | 0.644 | 0.751 | 0.239 | 0.038 | 0.978 | 0.093 |
|  |  | % | 2 | -5 | 3 | -1 | 3 | 4 | 3 | 8 | 1 | -6 | 6 | 10 | -1 | 9 |
| **Pathological diagnosis** | | | | | | | | | | | | | | | | |
|  |  |  | **AV** | **LD** | **LP** | **VA** | **VLa** | **VLp** | **VPL** | **VM** | **Intralaminar** | **Midline** | **MD** | **LGN** | **MGN** | **Pulvinar** |
| **FUS** | **tau-CBD** | *p-value* | 0.301 | 0.027 | 0.060 | 0.205 | 0.220 | 0.325 | 0.704 | 0.881 | 0.294 | 0.235 | 0.761 | 0.929 | 0.412 | 0.221 |
|  |  | % | 13 | 42 | 20 | 11 | 9 | 8 | 1 | 0 | 8 | 20 | 5 | 7 | -3 | 9 |
|  | **FTDP-17** | *p-value* | 0.965 | 0.169 | 0.246 | 0.837 | 0.621 | 0.263 | 0.055 | 0.127 | 0.279 | 0.603 | 0.521 | 0.012 | 0.265 | 0.178 |
|  |  | % | 2 | 29 | 13 | -1 | 3 | 6 | 10 | 8 | 5 | 5 | 6 | 22 | 7 | 10 |
|  | **tau-Pick's** | *p-value* | 0.785 | 0.193 | 0.650 | 0.775 | 0.741 | 0.819 | 0.711 | 0.717 | 0.734 | 0.814 | 0.792 | 0.678 | 0.749 | 0.426 |
|  |  | % | -3 | 23 | 5 | 2 | 2 | 2 | 2 | 0 | 1 | 5 | 5 | 1 | 1 | 5 |
|  | **tau-PSP** | *p-value* | 0.910 | 0.557 | 0.507 | 0.375 | 0.144 | 0.130 | 0.227 | 0.178 | 0.030 | 0.751 | 0.274 | 0.263 | 0.508 | 0.396 |
|  |  | % | 4 | *26* | 14 | 12 | 14 | 15 | 14 | 12 | 19 | 20 | 7 | 23 | 13 | 9 |
|  | **TDP-43 type A** | *p-value* | 0.152 | *0.009* | 0.020 | 0.433 | 0.588 | 0.584 | 0.863 | 0.682 | 0.136 | 0.174 | 0.696 | 0.170 | 0.852 | **0.001** |
|  |  | % | 15 | 45 | 23 | 7 | 4 | 5 | 3 | 4 | 8 | 20 | 10 | 14 | -1 | **23** |
|  | **TDP-43 type B** | *p-value* | 0.775 | 0.706 | 0.896 | 0.639 | 0.262 | 0.295 | 0.357 | 0.266 | 0.435 | 0.442 | 0.700 | 0.017 | 0.436 | 0.163 |
|  |  | % | -4 | -6 | -1 | 3 | 7 | 6 | 4 | 4 | 2 | -10 | -7 | 29 | 3 | 14 |
|  | **TDP-43 type C** | *p-value* | 0.231 | 0.599 | 0.488 | 0.087 | 0.178 | 0.383 | 0.931 | 0.866 | 0.777 | 0.143 | 0.023 | 0.348 | 0.503 | 0.243 |
|  |  | % | -10 | 16 | -2 | -8 | -5 | -2 | 1 | 0 | -1 | -5 | -13 | 14 | -4 | 8 |
| **tau-CBD** | **FTDP-17** | *p-value* | 0.234 | 0.329 | 0.400 | 0.077 | 0.385 | 0.846 | *0.005* | 0.041 | 0.944 | 0.429 | 0.249 | **0.003** | 0.019 | 0.853 |
|  |  | % | -13 | -22 | -10 | -12 | -6 | -2 | *8* | 8 | -3 | -19 | 1 | **16** | 9 | 1 |
|  | **tau-Pick's** | *p-value* | 0.059 | 0.138 | 0.032 | 0.141 | 0.176 | 0.257 | 0.287 | 0.475 | 0.281 | 0.154 | 0.420 | 0.485 | 0.101 | 0.476 |
|  |  | % | -18 | -33 | -19 | -9 | -7 | -7 | 1 | 0 | -7 | -19 | 0 | -6 | 3 | -5 |
|  | **tau-PSP** | *p-value* | 0.240 | 0.133 | 0.281 | 0.853 | 0.573 | 0.382 | 0.063 | 0.072 | 0.104 | 0.421 | 0.300 | 0.200 | 0.103 | 0.851 |
|  |  | % | -11 | -28 | -8 | 2 | 5 | 8 | 13 | 12 | 12 | 0 | 1 | 18 | 16 | 0 |
|  | **TDP-43 type A** | *p-value* | 0.638 | 0.696 | 0.630 | 0.445 | 0.301 | 0.501 | 0.431 | 0.437 | 0.596 | 0.884 | 0.330 | 0.080 | 0.352 | *0.008* |
|  |  | % | 3 | 6 | 3 | -4 | -5 | -4 | 2 | 4 | 0 | 0 | 5 | 8 | 2 | *15* |
|  | **TDP-43 type B** | *p-value* | 0.203 | 0.014 | 0.062 | 0.535 | 0.867 | 0.759 | 0.160 | 0.158 | 0.952 | 0.050 | 0.868 | *0.008* | 0.101 | 0.624 |
|  |  | % | -19 | -83 | -27 | -8 | -2 | -2 | 2 | 4 | -6 | -38 | -13 | *23* | 5 | 5 |
|  | **TDP-43 type C** | *p-value* | **0.001** | *0.009* | **<0.0005** | **<0.0005** | **<0.0005** | *0.007* | 0.649 | 0.642 | 0.047 | **<0.0005** | *0.006* | 0.233 | 0.766 | 0.838 |
|  |  | % | **-27** | *-44* | **-29** | **-21** | **-15** | *-11* | 0 | 0 | -9 | **-31** | *-19* | 8 | -1 | -2 |
| **FTDP-17** | **tau-Pick's** | *p-value* | 0.687 | 0.790 | 0.303 | 0.521 | 0.789 | 0.209 | 0.029 | 0.099 | 0.286 | 0.673 | 0.578 | **<0.0005** | 0.256 | 0.389 |
|  |  | % | -5 | -9 | -9 | 3 | -1 | -4 | -8 | -9 | -5 | 0 | -1 | **-27** | -7 | -6 |
|  | **tau-PSP** | *p-value* | 0.865 | 0.521 | 0.728 | 0.230 | 0.234 | 0.510 | 0.650 | 0.933 | 0.147 | 0.893 | 0.065 | 0.256 | 0.760 | 0.752 |
|  |  | % | 1 | -5 | 2 | 12 | 11 | 9 | 5 | 4 | 14 | 16 | 1 | 2 | 7 | -2 |
|  | **TDP-43 type A** | *p-value* | 0.086 | 0.157 | 0.176 | 0.213 | 0.992 | 0.411 | 0.019 | 0.124 | 0.686 | 0.320 | 0.707 | 0.091 | 0.086 | 0.027 |
|  |  | % | 13 | 23 | 11 | 7 | 1 | -2 | -8 | -4 | 3 | 16 | 4 | -10 | -8 | 14 |
|  | **TDP-43 type B** | *p-value* | 0.720 | 0.096 | 0.231 | 0.478 | 0.422 | 0.878 | 0.472 | 0.885 | 0.912 | 0.184 | 0.311 | 0.702 | 0.888 | 0.735 |
|  |  | % | -6 | -50 | -16 | 4 | 4 | 0 | -7 | -4 | -3 | -16 | -14 | 9 | -4 | 4 |
|  | **TDP-43 type C** | *p-value* | 0.117 | 0.214 | 0.014 | 0.061 | 0.018 | *0.009* | *0.006* | 0.058 | 0.065 | 0.011 | **<0.0005** | 0.021 | 0.018 | 0.696 |
|  |  | % | -13 | -18 | -17 | -7 | -8 | *-9* | *-10* | -9 | -6 | -11 | **-20** | -10 | -12 | -3 |
| **Tau - Pick's** | **tau-PSP** | *p-value* | 0.902 | 0.599 | 0.678 | 0.383 | 0.116 | 0.081 | 0.227 | 0.162 | 0.013 | 0.858 | 0.093 | 0.063 | 0.585 | 0.751 |
|  |  | % | 6 | 4 | 10 | 10 | 12 | 13 | 12 | 12 | 18 | 16 | 2 | 23 | 13 | 4 |
|  | **TDP-43 type A** | *p-value* | *0.006* | 0.029 | **0.002** | 0.413 | 0.724 | 0.598 | 0.756 | 0.930 | 0.060 | 0.067 | 0.830 | *0.004* | 0.416 | **<0.0005** |
|  |  | % | *18* | 29 | **18** | 5 | 2 | 3 | 1 | 4 | 7 | 16 | 5 | *13* | -1 | **19** |
|  | **TDP-43 type B** | *p-value* | 0.918 | 0.099 | 0.564 | 0.750 | 0.279 | 0.277 | 0.423 | 0.297 | 0.513 | 0.244 | 0.474 | **0.001** | 0.502 | 0.316 |
|  |  | % | -1 | -38 | -6 | 1 | 5 | 4 | 2 | 4 | 1 | -16 | -13 | **28** | 2 | 9 |
|  | **TDP-43 type C** | *p-value* | 0.113 | 0.185 | 0.051 | **0.001** | *0.004* | 0.060 | 0.435 | 0.742 | 0.286 | *0.004* | **<0.0005** | 0.021 | 0.091 | 0.519 |
|  |  | % | -7 | -8 | -8 | **-10** | *-7* | -4 | -1 | 0 | -1 | *-11* | **-19** | 13 | -5 | 3 |
| **Tau - PSP** | **TDP-43 type A** | *p-value* | 0.112 | 0.060 | 0.135 | 0.715 | 0.175 | 0.155 | 0.162 | 0.179 | 0.181 | 0.337 | 0.070 | 0.944 | 0.294 | 0.031 |
|  |  | % | 12 | 26 | 10 | -6 | -11 | -12 | -13 | -9 | -13 | 0 | 3 | -12 | -16 | 15 |
|  | **TDP-43 type B** | *p-value* | 0.863 | 0.351 | 0.445 | 0.699 | 0.767 | 0.674 | 0.801 | 0.843 | 0.186 | 0.290 | 0.504 | 0.196 | 0.894 | 0.572 |
|  |  | % | -8 | -43 | -18 | -10 | -8 | -10 | -12 | -9 | -20 | -38 | -15 | 7 | -12 | 5 |
|  | **TDP-43 type C** | *p-value* | 0.282 | 0.802 | 0.110 | **0.003** | **0.001** | *0.004* | 0.087 | 0.101 | **0.001** | 0.054 | 0.384 | 0.587 | 0.116 | 0.954 |
|  |  | % | -14 | -13 | -19 | **-23** | **-22** | *-20* | -15 | -14 | **-24** | -31 | -21 | -12 | -20 | -1 |
| **TDP-43 type A** | **TDP-43 type B** | *p-value* | 0.098 | *0.005* | 0.022 | 0.884 | 0.386 | 0.439 | 0.336 | 0.328 | 0.676 | 0.030 | 0.409 | 0.097 | 0.264 | 0.204 |
|  |  | % | -22 | *-94* | -30 | -4 | 3 | 2 | 1 | 0 | -6 | -38 | -19 | 17 | 3 | -12 |
|  | **TDP-43 type C** | *p-value* | **<0.0005** | **<0.0005** | **<0.0005** | **<0.0005** | **0.002** | 0.018 | 0.662 | 0.680 | **0.003** | **<0.0005** | **<0.0005** | 0.443 | 0.423 | **<0.0005** |
|  |  | % | **-30** | **-53** | **-32** | **-16** | **-10** | -7 | -2 | -4 | **-9** | **-31** | **-25** | 0 | -3 | **-19** |
| **TDP-43 type B** | **TDP-43 type C** | *p-value* | 0.454 | 0.342 | 0.634 | 0.032 | *0.009* | 0.036 | 0.222 | 0.221 | 0.219 | 0.691 | 0.106 | 0.035 | 0.114 | 0.509 |
|  |  | % | -6 | 21 | -2 | -11 | *-13* | -9 | -3 | -4 | -3 | 5 | -5 | -21 | -7 | -7 |
| **Clinical diagnosis** | | | | | | | | | | | | | | | | |
|  |  |  | **AV** | **LD** | **LP** | **VA** | **VLa** | **VLp** | **VPL** | **VM** | **Intralaminar** | **Midline** | **MD** | **LGN** | **MGN** | **Pulvinar** |
| **bvFTD** | **nfvPPA** | *p-value* | 0.893 | 0.210 | 0.478 | 0.059 | 0.136 | 0.137 | *0.009* | 0.014 | 0.381 | 0.845 | 0.068 | 0.012 | **<0.0005** | 0.044 |
|  |  | % | -1 | 4 | 0 | -2 | -1 | -1 | *-3* | 0 | 1 | 0 | -3 | -3 | **-4** | -3 |
|  | **PPA-NOS** | *p-value* | 0.166 | 0.682 | 0.224 | *0.004* | **0.003** | *0.006* | 0.078 | 0.098 | 0.107 | 0.071 | **0.003** | 0.152 | **0.003** | 0.141 |
|  |  | % | -6 | 4 | -5 | *-6* | **-6** | *-5* | -3 | 0 | -2 | -6 | **-10** | -4 | **-8** | -5 |
|  | **svPPA** | *p-value* | **<0.0005** | 0.090 | **0.001** | **<0.0005** | **<0.0005** | **<0.0005** | **<0.0005** | **0.001** | **0.001** | **<0.0005** | **<0.0005** | 0.471 | **<0.0005** | **<0.0005** |
|  |  | % | **-10** | -9 | **-8** | **-10** | **-8** | **-6** | **-6** | **-4** | **-5** | **-17** | **-11** | -1 | **-7** | **-11** |
|  | **FTD-MND** | *p-value* | 0.020 | 0.028 | **0.002** | 0.459 | 0.392 | 0.163 | 0.650 | 0.927 | 0.071 | *0.008* | 0.116 | 0.700 | 0.936 | 0.238 |
|  |  | % | 17 | 35 | **24** | 4 | 4 | 5 | 1 | 0 | 6 | *17* | 10 | 5 | 1 | 8 |
| **nfvPPA** | **PPA-NOS** | *p-value* | 0.158 | 0.867 | 0.132 | 0.052 | 0.028 | 0.047 | 0.593 | 0.616 | 0.049 | 0.065 | 0.035 | 0.794 | 0.184 | 0.597 |
|  |  | % | -4 | 0 | -5 | -4 | -4 | -4 | 0 | 0 | -3 | -6 | -6 | -1 | -3 | -2 |
|  | **svPPA** | *p-value* | **<0.0005** | *0.009* | **<0.0005** | **<0.0005** | **<0.0005** | **0.001** | 0.272 | 0.410 | **<0.0005** | **<0.0005** | **0.001** | 0.134 | 0.479 | **0.003** |
|  |  | % | **-9** | *-14* | **-8** | **-8** | **-6** | **-5** | -3 | -4 | **-6** | **-17** | **-8** | 3 | -3 | **-7** |
|  | **FTD-MND** | *p-value* | 0.024 | 0.080 | *0.004* | 0.169 | 0.176 | 0.059 | 0.180 | 0.454 | 0.136 | 0.010 | 0.030 | 0.217 | 0.194 | 0.064 |
|  |  | % | 18 | 32 | *24* | 6 | 5 | 6 | 4 | 0 | 6 | 17 | 13 | 8 | 5 | 11 |
| **PPA-NOS** | **svPPA** | *p-value* | 0.418 | 0.233 | 0.632 | 0.570 | 0.899 | 0.880 | 0.966 | 0.947 | 0.939 | 0.609 | 0.679 | 0.302 | 0.346 | 0.316 |
|  |  | % | -4 | -14 | -3 | -4 | -2 | -1 | -2 | -4 | -3 | -11 | -1 | 4 | 1 | -6 |
|  | **FTD-MND** | *p-value* | *0.006* | 0.117 | **0.001** | 0.018 | 0.012 | *0.005* | 0.145 | 0.347 | 0.013 | **0.001** | **0.002** | 0.232 | 0.055 | 0.060 |
|  |  | % | *21* | 32 | **28** | 10 | 9 | *10* | 4 | 0 | 8 | **21** | **18** | 9 | 8 | 12 |
| **svPPA** | **FTD-MND** | *p-value* | **<0.0005** | *0.006* | **<0.0005** | **0.001** | **0.002** | **0.001** | 0.079 | 0.287 | **0.003** | **<0.0005** | **0.001** | 0.525 | 0.118 | **0.003** |
|  |  | % | **24** | *40* | **30** | **13** | **10** | **11** | 6 | 4 | **11** | **29** | **20** | 5 | 7 | **17** |

**Supplementary Table 2. Volumetric comparisons for the thalamic nuclei between the clinical subgroups with sporadic FTD and the controls.** Volumetric comparisons, expressed as % of TIV, are adjusted for age, gender, TIV and scanner type. Bold represents a significant difference between groups after correcting for multiple comparisons.

|  | **n** |  | **AV** | **LD** | **LP** | **VA** | **VLa** | **VLp** | **VPL** | **VM** | **Intralaminar** | **Midline** | **MD** | **LGN** | **MGN** | **Pulvinar** |
| --- | --- | --- | --- | --- | --- | --- | --- | --- | --- | --- | --- | --- | --- | --- | --- | --- |
| **Controls** | **104** | **Mean** | 0.017 | 0.003 | 0.015 | 0.054 | 0.079 | 0.103 | 0.109 | 0.003 | 0.051 | 0.002 | 0.126 | 0.021 | 0.016 | 0.025 |
|  |  | **SD** | 0.002 | 0.001 | 0.002 | 0.004 | 0.006 | 0.008 | 0.011 | 0.000 | 0.004 | 0.000 | 0.015 | 0.003 | 0.002 | 0.004 |
| **FTD-MND** | **4** | **Mean** | 0.012 | 0.002 | 0.010 | 0.046 | 0.068 | 0.087 | 0.098 | 0.002 | 0.044 | 0.002 | 0.086 | 0.018 | 0.015 | 0.023 |
|  |  | **SD** | 0.001 | 0.000 | 0.002 | 0.006 | 0.009 | 0.010 | 0.008 | 0.000 | 0.004 | 0.000 | 0.005 | 0.002 | 0.001 | 0.005 |
| **bvFTD** | **120** | **Mean** | 0.014 | 0.002 | 0.012 | 0.047 | 0.071 | 0.093 | 0.101 | 0.002 | 0.047 | 0.002 | 0.095 | 0.019 | 0.014 | 0.025 |
|  |  | **SD** | 0.003 | 0.001 | 0.003 | 0.006 | 0.007 | 0.009 | 0.011 | 0.000 | 0.005 | 0.000 | 0.016 | 0.003 | 0.002 | 0.004 |
| **nfvPPA** | **106** | **Mean** | 0.014 | 0.002 | 0.012 | 0.049 | 0.072 | 0.094 | 0.102 | 0.002 | 0.047 | 0.002 | 0.098 | 0.019 | 0.015 | 0.025 |
|  |  | **SD** | 0.003 | 0.001 | 0.003 | 0.006 | 0.007 | 0.009 | 0.010 | 0.000 | 0.005 | 0.000 | 0.016 | 0.003 | 0.002 | 0.004 |
| **PPA-NOS** | **13** | **Mean** | 0.015 | 0.002 | 0.013 | 0.049 | 0.073 | 0.096 | 0.101 | 0.002 | 0.047 | 0.002 | 0.101 | 0.020 | 0.015 | 0.026 |
|  |  | **SD** | 0.003 | 0.001 | 0.002 | 0.005 | 0.006 | 0.007 | 0.012 | 0.000 | 0.004 | 0.000 | 0.021 | 0.003 | 0.002 | 0.004 |
| **svPPA** | **85** | **Mean** | 0.015 | 0.003 | 0.013 | 0.053 | 0.076 | 0.099 | 0.105 | 0.003 | 0.049 | 0.002 | 0.105 | 0.019 | 0.015 | 0.027 |
|  |  | **SD** | 0.003 | 0.001 | 0.003 | 0.005 | 0.007 | 0.009 | 0.011 | 0.000 | 0.005 | 0.000 | 0.018 | 0.003 | 0.002 | 0.003 |
| **Controls** | **FTD-MND** | *p-value* | **0.002** | 0.060 | **0.001** | 0.018 | 0.019 | 0.005 | 0.042 | 0.281 | 0.014 | **<0.0005** | **<0.0005** | 0.361 | 0.450 | 0.378 |
|  |  | % | **29** | 41 | **33** | 15 | 13 | 15 | 11 | 8 | 14 | **35** | **31** | 14 | 7 | 10 |
|  | **bvFTD** | *p-value* | **<0.0005** | **<0.0005** | **<0.0005** | **<0.0005** | **<0.0005** | **<0.0005** | **<0.0005** | **<0.0005** | **<0.0005** | **<0.0005** | **<0.0005** | **0.002** | **<0.0005** | 0.650 |
|  |  | % | **16** | **28** | **18** | **12** | **10** | **9** | **8** | **8** | **8** | **17** | **25** | **8** | **11** | 2 |
|  | **nfvPPA** | *p-value* | **<0.0005** | **<0.0005** | **<0.0005** | **<0.0005** | **<0.0005** | **<0.0005** | **<0.0005** | **0.003** | **<0.0005** | **<0.0005** | **<0.0005** | 0.045 | **0.003** | 0.392 |
|  |  | % | **16** | **28** | **16** | **9** | **8** | **8** | **6** | **8** | **9** | **22** | **22** | 7 | **6** | -1 |
|  | **PPA-NOS** | *p-value* | 0.023 | **0.002** | 0.026 | 0.010 | 0.042 | 0.066 | 0.034 | 0.073 | 0.017 | **0.002** | **<0.0005** | 0.369 | 0.539 | 0.527 |
|  |  | % | 11 | **28** | 12 | 9 | 7 | 6 | 8 | 8 | 8 | **17** | **20** | 6 | 5 | -2 |
|  | **svPPA** | *p-value* | **<0.0005** | **<0.0005** | **<0.0005** | 0.080 | 0.059 | 0.011 | 0.005 | 0.027 | **0.001** | **<0.0005** | **<0.0005** | **<0.0005** | 0.009 | **0.001** |
|  |  | % | **9** | **22** | **11** | 3 | 3 | 4 | 4 | 4 | **4** | **9** | **17** | **10** | 4 | **-7** |

**Supplementary Table 3.** **Volumetric comparisons for the thalamic nuclei between the pathological subgroups with sporadic FTD and the controls.** Volumes are expressed as % of total intracranial volume (TIV).

|  |  | **AV** | **LD** | **LP** | **VA** | **VLa** | **VLp** | **VPL** | **VM** | **Intralaminar** | **Midline** | **MD** | **LGN** | **MGN** | **Pulvinar** |
| --- | --- | --- | --- | --- | --- | --- | --- | --- | --- | --- | --- | --- | --- | --- | --- |
| **TDP-43 type A** | **Mean** | 0.015 | 0.003 | 0.012 | 0.052 | 0.078 | 0.101 | 0.107 | 0.003 | 0.048 | 0.002 | 0.095 | 0.018 | 0.015 | 0.025 |
|  | **SD** | 0.002 | 0.001 | 0.002 | 0.003 | 0.003 | 0.003 | 0.010 | 0.000 | 0.010 | 0.000 | 0.023 | 0.003 | 0.002 | 0.004 |
|  | **%** | 13 | 16 | 16 | 4 | 1 | 1 | 2 | 0 | 6 | 22 | 24 | 12 | 6 | 2 |
| **TDP-43 type B** | **Mean** | 0.013 | 0.003 | 0.011 | 0.046 | 0.066 | 0.086 | 0.104 | 0.002 | 0.044 | 0.002 | 0.104 | 0.018 | 0.015 | 0.024 |
|  | **%** | 22 | 9 | 27 | 15 | 16 | 16 | 5 | 8 | 14 | 17 | 17 | 12 | 2 | 5 |
